# NEP-Score Thresholds Predict Survival of Patients With Bronchial Carcinoids

**DOI:** 10.3389/fendo.2020.621557

**Published:** 2021-02-08

**Authors:** Irene Gagliardi, Mario Tarquini, Maria Rosaria Ambrosio, Elisa Giannetta, Patricia Borges de Souza, Roberta Gafà, Aldo Carnevale, Paola Franceschetti, Maria Chiara Zatelli

**Affiliations:** ^1^ Section of Endocrinology and Internal Medicine, Department of Medical Sciences, University of Ferrara, Ferrara, Italy; ^2^ Endocrine Unit, Azienda Ospedaliero-Universitaria di Ferrara, Ferrara, Italy; ^3^ Section of Medical Physiopathology, Department of Experimental Medicine, Sapienza University of Rome, Rome, Italy; ^4^ Pathology Unit, Department of Translational Medicine, University of Ferrara, Ferrara, Italy; ^5^ Department of Translational Medicine, University of Ferrara, Ferrara, Italy

**Keywords:** bronchial neuroendocrine neoplasms, NEP-Score, NEP-D, NEP-T, Delta NEP score, survival

## Abstract

Survival prognostic markers are extremely needed to better define therapeutic strategies in patients with bronchial carcinoids (BC). We aim to verify the applicability of the NEP-Score in a homogeneous BC cohort and identify a derivative prognostic marker, the NEP-Score at diagnosis (NEP-D) that does not consider new metastases during follow-up. Sixty-four patients (38 females, and 26 males, mean age at diagnosis 58.9 ± 1.7 years) with BC were retrospectively evaluated. NEP-Score was calculated at the end of follow-up (NEP-T). A derivative score, the NEP-Score at diagnosis (NEP-D) that does not consider new metastases during follow-up, was then assessed. Patients were subdivided according to their living status at the end of follow-up. A NEP-Score threshold was investigated to predict survival. Mean NEP-T and mean NEP-D were significantly lower in live patients at end of follow-up. A NEP-T cut-off >138 significantly predicts survival. Atypical BC relapsed more frequently than Typical BC. Male gender and previous malignancy were negative prognostic factors for survival. We confirmed NEP-Score applicability in BC and NEP-D utility, being the latter a simple, quick, and cheap prognostic score that can help clinicians in decision making. The identified NEP-D threshold can predict NEN aggressiveness and may be used to define the best personalized therapeutic strategy. In this context, a validation study is needed.

## Introduction

Bronchial carcinoids (BC) are uncommon bronchial/pulmonary tumors characterized by a wide spectrum of clinical behavior, representing 20%–30% of all neuroendocrine neoplasms (NEN), whose incidence rates range from 0.2 to 2/100.000 people/year, and most series suggest a higher incidence in women as compared to men and in Caucasian as compared to black patients (1–6). In the United States Surveillance, Epidemiology, and End Results database, bronchial NEN annual incidence between 2000 and 2012 was 1.49 per 100,000 population (6). Several reports suggest that BC incidence is increasing over time (2, 3, 6); this may be at least partly related to the increased use of advanced medical imaging techniques that detect a higher number of asymptomatic tumors. Mean age at diagnosis for Typical BC (TBC) is 45 years, while in many series, patients with Atypical BC (ABC) are ~10 years older (7, 8), possibly influencing prognosis. BC management is mainly influenced by tumor differentiation and Ki-67 (9–14), but markers of clinical outcomes that could predict patient survival are still lacking (15–19). Despite improvements in prognostic grading and staging systems, the challenge to predict BC patient’s outcome is difficult. This is particularly true for patients with ABC, since they often have a worse prognosis with a greater tendency to metastasize and recur locally (20), with more frequent distant metastases (liver or bone) more frequent as compared to local recurrence (20). In addition, nodal metastases have an adverse influence on ABC prognosis, that is worse as compared to TBC. On the contrary, TBC usually have an excellent prognosis following surgical resection and the prognostic impact of nodal involvement remains controversial. Overall, BC may display a wide spectrum of clinical behavior, ranging from indolent to aggressive, with few validated prognostic factors that could help clinicians to predict survival. Several putative markers have been considered, including blood-based biomarkers, such as Chromogranin A [CgA] (21), circulating tumor cells and microRNAs (22), as well as tissue markers (23), but none has been fully validated, so far. Pusceddu and co-authors (24) developed a classification prognostic score for overall survival (OS) in patients with well differentiated NEN, named NEuroendocrine Prognostic Score (NEP-Score], that we recently validated in an independent cohort of entero-pancreatic NEN (25). In the study by Pusceddu (24), NEP-Score turned out to be useful to rank patients according to their mortality risk and to predict OS. The aim of the present study is to verify NEP-Score applicability in a homogenous BC cohort and to identify a derivate marker capable to predict patients’ prognosis by taking into account clinical and pathological characteristics at diagnosis. Furthermore, we considered the impact of other prognostic factors, such as gender, differentiation, and previous malignancy.

## Materials and Methods 

### Study Design

We retrospectively evaluated the NEP-Score in a series of BC patients referring to our center from 1992 to 2018. According to Pusceddu S. et al. (24), only patients with a >24 months follow-up with TBC or ABC with a Ki-67 index 0%–2% and 3%–20%, respectively, were included, while patients with poorly-differentiated neuroendocrine carcinomas were excluded.

### Patients

We collected data on 64 patients (38 females, 26 males; mean age at diagnosis 58.9 ± 1.7 years) including seven ABC and 57 TBC followed up in our center from 1992 to 2018. Patients were evaluated for the following characteristics to calculate the NEP-Score at the end of follow-up (NEP-T) which lasted 98.2±11.3 months: age, site of primary tumor, primary tumor surgery, symptoms, Ki-67, timing of metastases, assigning the respective scores [see **Table 1**]. A modified NEP-Score at diagnosis (NEP-D), which does not take into account the appearance of new metastases during follow-up, was then calculated. Patients were subdivided according to their vital status (alive or not) at the end of follow-up (EOF). We also considered the difference between NEP-T score and NEP-D score, indicated as Delta NEP, in order to appropriately evaluate progression during follow-up. We considered as positive a Delta NEP>0, and as negative a Delta NEP=0. Patients characteristics are displayed in **Table 2**. This study is in accordance with the principles set out in the Declaration of Helsinki, has been specifically approved by the Local Ethics Committee (Comitato Etico Indipendente di Area Vasta Emilia Centro, CE-AVEC, at the Policlinico S. Orsola-Malpighi in Bologna) and authorized by the General Director of the Azienda Ospedaliero Universitaria in Ferrara (protocol number CE-AVEC 238/2020/Oss/AOUFe). Each patient has been informed of the purpose and nature of all procedures used.

**Table 1:** NEP-Score calculation (modified from 24).

	Score
**Age**	
<45	0
46–65	28
>65	58
**Site of primary tumor**	
Bronchial	72
**Primary tumor surgery**	
Yes	0
No	100
**Functional status**	
Yes	32
No	0
**Ki-67**	
0–2	0
3–20	12
**Timing of metastases**	
Synchronous	0
Metachronous > 24 months	38
Metachronous ≤ 24 months	72

**Table 2:** Patients characteristics.

n°	Gender	Site of primary tumor	Age at diagnosis	Ki67	syndrome	Primary tumor surgery	Timing of metastasis	NEP-D	NEP-T	Dead
1	F	Bronchial	63	0-2%	No	Yes	No	100	100	Yes
2	F	Bronchial	70	Missing	No	Yes	Meta > 24 months	130	168	Yes
3	F	Bronchial	64	Missing	No	Yes	No	100	100	Yes
4	F	Bronchial	58	Missing	No	Yes	No	100	100	Yes
5	F	Bronchial	69	0-2%	No	Yes	No	130	130	No
6	F	Bronchial	44	0%–2%	No	Yes	No	72	72	No
7	F	Bronchial	39	0%–2%	No	Yes	No	72	72	No
8	F	Bronchial	55	3%–20%	No	Yes	No	112	112	No
9	F	Bronchial	37	Missing	No	Yes	No	72	72	No
10	F	Bronchial	77	Missing	No	Yes	No	130	130	No
11	F	Bronchial	72	0%–2%	No	Yes	No	130	130	No
12	F	Bronchial	31	Missing	No	Yes	No	72	72	No
13	F	Bronchial	56	0%–2%	No	Yes	No	100	100	No
14	F	Bronchial	42	Missing	No	Yes	No	72	72	No
15	F	Bronchial	70	0%–2%	No	Yes	No	130	130	No
16	F	Bronchial	77	0%–2%	No	Yes	Meta > 24 months	130	168	No
17	F	Bronchial	53	Missing	No	Yes	Meta < 24 months	100	172	No
18	F	Bronchial	69	Missing	No	Yes	No	130	130	No
19	F	Bronchial	51	3%–20%	No	Yes	Meta < 24 months	112	184	No
20	F	Bronchial	51	0%–2%	No	Yes	No	100	100	No
21	F	Bronchial	68	0%–2%	No	Yes	No	130	130	No
22	F	Bronchial	52	0%–2%	No	Yes	No	100	100	No
23	F	Bronchial	60	0%–2%	No	Yes	No	100	100	No
24	F	Bronchial	60	0%–2%	No	Yes	No	100	100	No
25	F	Bronchial	36	0%–2%	No	Yes	No	72	72	No
26	F	Bronchial	57	3%–20%	No	Yes	No	112	112	No
27	F	Bronchial	71	0%–2%	No	Yes	No	130	130	No
28	F	Bronchial	69	0%–2%	No	Yes	No	130	130	No
29	F	Bronchial	47	0%–2%	No	Yes	No	100	100	No
30	F	Bronchial	57	0%–2%	No	Yes	No	100	100	No
31	F	Bronchial	70	0%–2%	No	Yes	No	130	130	No
32	F	Bronchial	75	3%–20%	No	Yes	No	142	142	No
33	F	Bronchial	59	0%–2%	No	Yes	No	100	100	No
34	F	Bronchial	40	0%–2%	No	Yes	No	72	72	No
35	F	Bronchial	61	0%–2%	No	Yes	No	100	100	No
36	F	Bronchial	63	0%–2%	No	Yes	No	100	100	No
37	F	Bronchial	47	0%–2%	No	Yes	No	100	100	No
38	F	Bronchial	65	0%–2%	No	Yes	No	100	100	No
39	M	Bronchial	46	Missing	No	Yes	Meta > 24 months	100	138	Yes
40	M	Bronchial	80	0%–2%	No	Yes	No	130	130	Yes
41	M	Bronchial	73	3%–20%	No	Yes	Meta < 24 months	142	214	Yes
42	M	Bronchial	72	3%–20%	No	Yes	Meta > 24 months	142	180	Yes
43	M	Bronchial	46	0%–2%	No	Yes	Meta > 24 months	100	138	Yes
44	M	Bronchial	80	0%–2%	No	Yes	No	130	130	Yes
45	M	Bronchial	64	Missing	No	Yes	Meta < 24 months	100	172	Yes
46	M	Bronchial	79	0%–2%	No	Yes	No	130	130	Yes
47	M	Bronchial	63	3%–20%	No	Yes	Meta < 24 months	112	184	Yes
48	M	Bronchial	70	0%–2%	No	Yes	Meta < 24 months	130	202	Yes
49	M	Bronchial	74	3%–20%	No	Yes	No	142	142	Yes
50	M	Bronchial	56	0%–2%	No	Yes	Meta > 24 months	100	138	No
51	M	Bronchial	58	Missing	No	Yes	No	100	100	No
52	M	Bronchial	63	Missing	No	Yes	No	100	100	No
53	M	Bronchial	31	3%–20%	No	Yes	No	84	84	No
54	M	Bronchial	72	0%–2%	No	Yes	Meta > 24 months	130	168	No
55	M	Bronchial	45	Missing	No	Yes	Meta > 24 months	72	110	No
56	M	Bronchial	57	Missing	No	Yes	Meta > 24 months	112	150	No
57	M	Bronchial	75	0%–2%	No	Yes	No	130	130	No
58	M	Bronchial	72	3%–20%	No	Yes	No	142	142	No
59	M	Bronchial	54	0%–2%	No	Yes	No	100	100	No
60	M	Bronchial	34	0%–2%	No	Yes	No	72	72	No
61	M	Bronchial	48	0%–2%	No	Yes	No	100	100	No
62	M	Bronchial	59	0%–2%	No	Yes	No	100	100	No
63	M	Bronchial	22	0%–2%	Yes	Yes	Meta > 24 months	104	142	No
64	M	Bronchial	71	0%–2%	No	Yes	No	130	130	No

### Statistical Evaluation

Categorical data were summarized using frequencies and percentages. The chi-square test was performed to evaluate the presence of statistically significant differences among the evaluated groups in terms of NEP-Score. The paired Student t-test was employed to compare mean NEP-D and NEP-T scores among groups. A p-value <0.05 was considered significant. Sensitivity, specificity, positive predictive value (PPV), negative predictive value (NPV), and accuracy were calculated for each identified NEP-D and NEP-T threshold. Statistical analysis was performed using GraphPad Prism version 6.01 for Windows, GraphPad Software, San Diego, California USA, www.graphpad.com.

## Results

### NEP Scores

#### NEP-T Score Calculation

Among the 64 evaluated patients, 49 were alive at EOF, seven died due to other causes (DOC) and 8 died due specifically for NEN (DNEN). Mean NEP-T, corresponding to the original NEP-Score, was 121.2 ± 4.2. Patients were then subdivided according to whether they were alive or not at EOF. We found that NEP-T in living patients (112.8 ± 4.1) did not significantly differ from that of DOC patients (122.6 ± 8.8; p= n.s.] but was significantly lower as compared to DNEN patients (171.2 ± 10.4; p<0.01 vs. live patients and p<0.01 vs. DOC patients). Therefore, NEP-T seems to be useful as a prognostic score in BC, taking into account the cause of death.

#### NEP-D Score Calculation

A modified NEP-Score was then calculated, considered as the NEP-Score at diagnosis (NEP-D), which does not take into account the appearance of new metastases during follow-up. Mean NEP-D was 108.5 ± 2.6. Patients were subdivided according to whether they were alive or not at EOF. We found that mean NEP-D in live patients (105.2 ± 3) was lower as compared to DOC (117 ± 5.7) and DNEN patients (121 ± 7.1), but these differences were not statistically significant. Therefore, NEP-D does not seem to be useful as a prognostic score in BC in our settings.

### Gender

**Figure 1:**
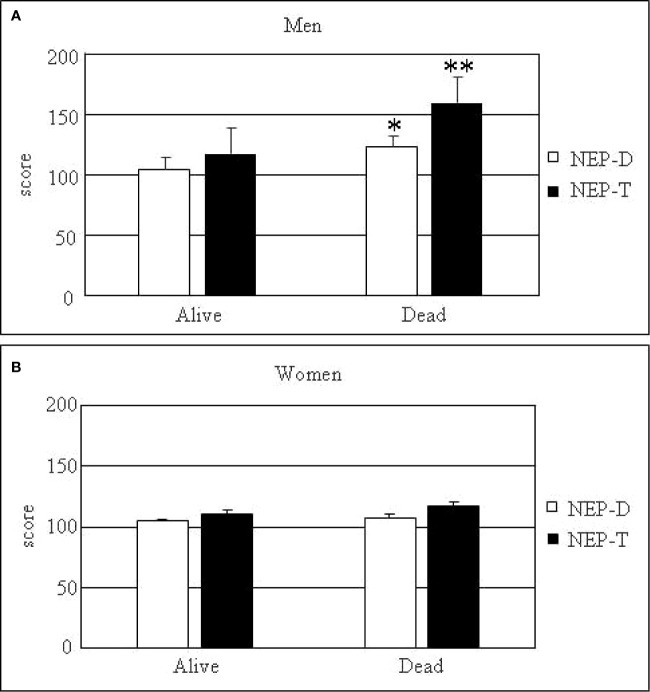
NEP-T and NEP-D scores according to gender. NEP-T (black columns) and NEP-D (white columns) scores are expressed as mean ± standard error of the mean (SEM). *p < 0.05 and **p < 0.01 dead vs. alive patients at the end of follow-up. **(A)** Men; **(B)** Women.

We also considered a possible difference between genders. In males, mean NEP-T of the 15 patients (mean age at EOF = 65.7 ± 4.4 years) who were alive at EOF was significantly lower as compared to that of the 11 patients (mean age at EOF = 75.2 ± 3.3 years) who were dead at EOF (117.7 ± 9.4; p<0.01) (**Figure 1A**). This difference was not correlated to aging, since mean age at EOF in the two groups was not significantly different. In females, mean NEP-T of the 33 patients (mean age at EOF= 64.7 ± 2.2 years) who were alive at EOF was similar to that of the four patients (mean age at EOF = 71.4 ± 5.3) who were dead at EOF (110.6 ± 5.2 vs. 117 ± 17; p = not significant) (**Figure 1B**). Comparing males and females (dead and alive at EOF), we found that mean NEP-T was significantly higher in males as compared to females (135 ± 6.9 vs. 111.3 ± 4.8; p<0.01). Mean NEP-T in DOC patients was similar in males and females (130 vs. 117 ± 17). DOC patients represented 10.5% of females (four out of 38) and 11.5% of male patients (3 out of 26). None of the female patients died due to BC progression, whereas DNEN represented 30.7% of male patients. NEP-T of the eight male DNEN patients was significantly higher as compared to the NEP-T of the 3 male DOC patients (171.3 ± 10.4 vs. 130; p<0.05). Therefore, NEP-T mirrors NEN-related prognosis and is not useful as a prognostic score in DOC patients in our settings.

Concerning NEP-D, as shown in **Figure 1A**, in male patients mean NEP-D of the 15 patients (mean age at diagnosis = 54.4 ± 4.1 years) who were alive at EOF was significantly lower as compared to that of the 11 patients (mean age at diagnosis = 67.9 ± 3.6 years) who were dead at EOF (105 ± 5.3 vs. 123 ± 5.2; p<0.05). This difference could be correlated to aging, since mean age at diagnosis in the two groups was significantly different (p<0.05). As shown in **Figure 1B**, in female patients mean NEP-D of the 33 patients (mean age at diagnosis = 57.3 ± 2.2 years) who were alive at EOF was similar to that of the four patients (mean age at diagnosis = 63.7 ± 2.4 years) who were dead at EOF (105.1 ± 3.8 vs. 107.5 ± 7.5; p= not significant). Comparing males and females, we found that mean NEP-D was similar in all groups, taking into account the influence of aging.

**Figure 2:**
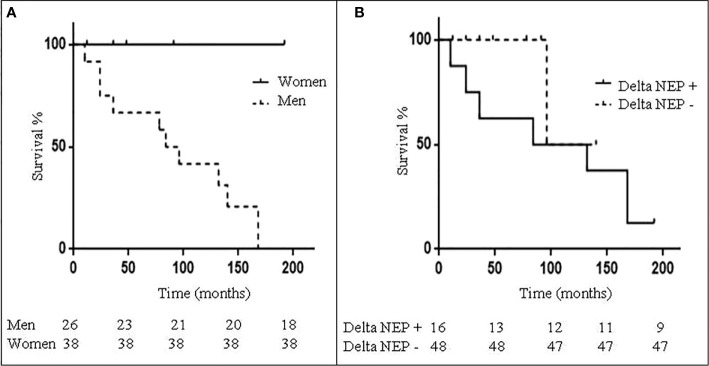
Patients survival according to gender and Delta NEP. **(A)** Kaplan-Meyer curves showing survival of 64 bronchial carcinoid (BC) patients according to gender; female are represented by the continuous line and males by the dotted line. **(B)** Kaplan-Meyer curves showing survival of 64 BC patients according to Delta NEP score; patients with positive Delta NEP score are represented by the continuous line and those with negative Delta NEP score by the dotted line.

Females had a better survival as compared to males, when taking into account only DNEN patients (**Figure 2A**). In addition, mean NEP-D was similar in males (112.8 ± 4.1) and in females (105.6 ± 3.4), while mean NEP-T was significantly higher in males as compared to females (135.6 ± 6.9 vs. 111.3 ± 4.8; p<0.005). Interestingly, mean Delta NEP was significantly higher in males as compared to females (22.7 ± 5.4 vs. 5.8 ± 2.9; p<0.005), suggesting that disease progression/recurrence is more likely to occur in males as compared to females. Indeed, a higher number of male patients displayed a positive Delta NEP as compared to females (p<0.01). Indeed, male patients relapsed more frequently as compared to females (46.1% vs. 10.5%; p<0.01), with a consequently higher NEN-related mortality in males as compared to females (30.7% vs. none). As shown in **Figure 2B**, Delta NEP positivity was associated with a worse survival. These data suggest that male gender may represent a negative prognostic factor in BC, in keeping with previous reports (15–17).

### NEP-T Threshold

**Figure 3:**
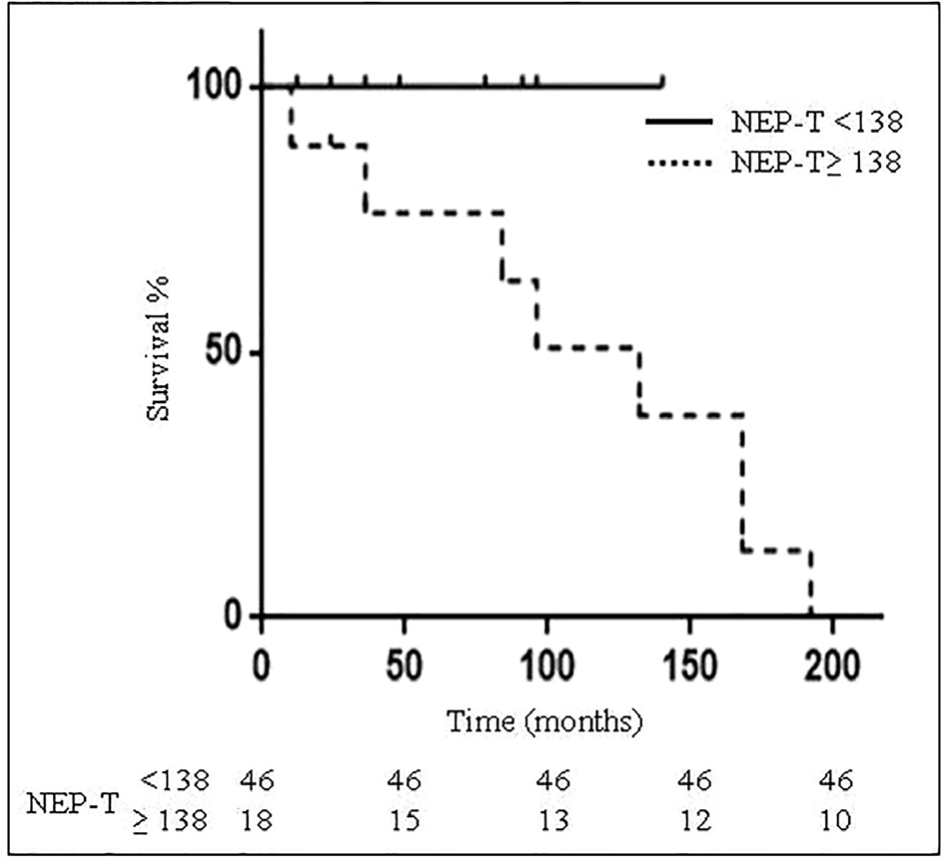
Patients survival according to NEP-T threshold. Kaplan-Meyer curves for survival of 64 bronchial carcinoid (BC) patients according to NEP-T threshold <138 (continuous line) or ≥138 (dotted line).

A NEP-T threshold was investigated to assess the reliability of this score to detect disease status. We found that a NEP-T score cut-off ≥138 could correctly differentiate patients alive from those dead at EOF. Indeed, 8 out of 18 patients with NEP-T ≥ 138 and none of 46 patients with NEP-T <138 were DNEN at EOF (**Figure 3**). A NEP-T≥ 138 threshold represents a cut-off allowing the best compromise between sensitivity (100%) and specificity (83.7%), with a PPV = 50%, a NPV = 100%, and accuracy = 85.9% to predict survival (dead vs. alive at EOF specifically for NEN). In addition, we found a higher recurrence rate in patients with NEP-T≥138 as compared to patients with NEP-T<138 (83.3% vs. 2.2%; p<0.01). Furthermore, patients with ABC were significantly more represented among patients with NEP-T≥138 as compared to patients with NEP-T<138 (23.5% vs. 4.2%; p<0.05).

### NEP-D Threshold

**Figure 4:**
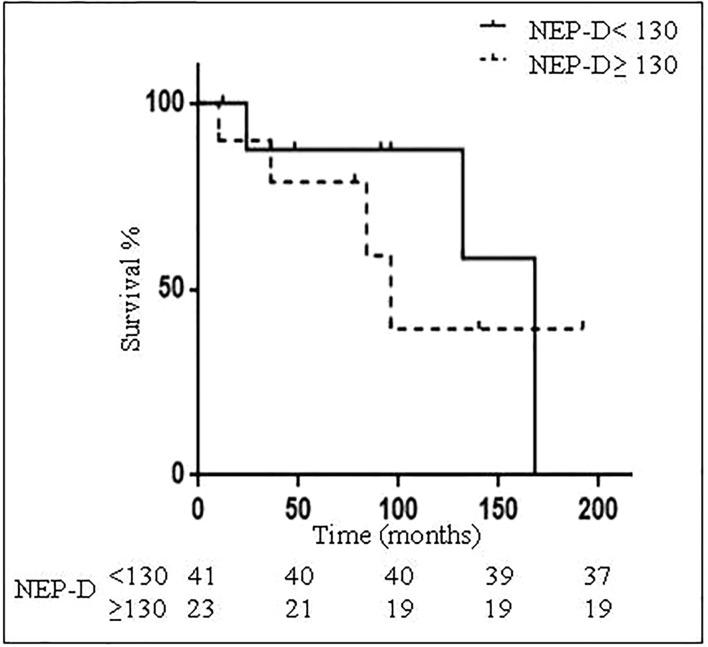
Patients survival according to NEP-D threshold. Kaplan-Meyer curves for survival of 64 bronchial carcinoid (BC) patients according to NEP-D threshold <130 (continuous line) or ≥ 130 (dotted line).

A NEP-D score threshold was investigated to predict survival at diagnosis. We found that a value of NEP-D≥ 130 represents a cut-off which allows for the best compromise between sensitivity (50%) and specificity (69.4%), with a PPV = 21%, a NPV = 89.5%, and accuracy = 66.7% to predict outcome (dead vs. alive at EOF). At EOF DNEN patients represented 17.4% of patients with NEP-D ≥ 130 and 9.7% of patients with NEP-D <130. However, statistical evaluation did not show any significant difference. Mean OS was 88.4±15.4 months and it was shorter in patients with NEP-D≥ 130 as compared to patients with NEP-D< 130 (56.5 ± 14.3 vs. 123 ± 25.7 months; p = not significant) (**Figure 4**). Patients who were alive at EOF showed a significantly lower mean Delta NEP score as compared to DOC (7.6±2.6 vs. 29.3±8; p<0.01) and DNEN patients (50.2±9.3; p<0.01). In addition, 85.4% of patients with negative Delta NEP were alive vs. 50% of patients with positive Delta NEP (p<0.05), suggesting that Delta NEP score positivity is associated with higher mortality.

### Patients Survival

During follow up (98.2±11.3 months) 15 patients died, with an overall mortality of 23.4% from other causes (DOC) and 12.5% specifically for NEN (DNEN) and similar OS (88.4±15.4 months for DOC and 89.7±22.2 months for DNEN). We then investigated whether the identified NEP-D threshold may be useful to predict 5-year survival: 75% of patients with NEP-D <130 were alive 5 years after diagnosis, whereas 46.7% of patients with NEP-D score ≥130 were alive 5 years after diagnosis. However, the observed different distribution did not reach statistical significance, probably due to the small number of enrolled patients. On the other hand, OS was significantly higher in patients with NEP-D <130 as compared to those with NEP-D ≥ 130 (161.1 ± 20.8 months vs. 93.6 ± 15.6 months; p= 0.05). Considering DNEN patients only, 5-years progression free survival (PFS) was longer in patients with NEP-D<130 as compared to patients with NEP-D≥130 (120.5±21.2 vs. 83±15.2 months). However, this difference did not reach statistical significance, probably due to the small sample size.

### Disease Recurrence

Recurrence was significantly more frequent among males vs. females (46% vs. 10.5%; p<0.05) and in patients who were dead as compared to those who were alive at EOF (87.5% vs. 19.5%; p<0.005). During follow-up, patients with ABC had a greater recurrence rate as compared to TBC (57.1% vs. 21%; p<0.05).

### Second Malignancy

Eight out of 64 patients (12.5%) developed a second malignancy during follow-up: 1 DOC and 1 DNEN. Patients with second malignancy had a significantly higher mean NEP-T as compared to patients without a second malignancy (147 ± 11.2 vs. 116.9 ± 4.4; p<0.05).

## Discussion

Our results show that the NEP-Score, corresponding to the NEP-T score in our study, is a valuable prognostic tool in BC management, since higher NEP-T scores associated with a worse survival. Therefore, we provide further validation of the NEP-score in a homogenous cohort of BC, after demonstrating its applicability in a homogeneous cohort of stage IV entero-pancreatic NEN (25). The possibility to identify a score that could predict BC prognosis is of great interest for clinicians in order to improve patient management. In these settings, several therapeutic approaches, including surgery, medical therapy with somatostatin analogs (SSA) (26) or everolimus (27–29) and PRRT (30) are indeed available. Scant data are available concerning efficacy of loco-regional approaches (chemoembolization, radioembolization, radiofrequency ablation) in BC (31). Since randomized studies are scanty and the disease is relatively uncommon the level of evidence is limited as compared to more common cancers (32). Therefore, an optimal management of the disease is difficult to establish. Thus, it would be very important to identify those patients that may benefit of an aggressive treatment from those who could be spared from unnecessary therapy. Consolidated prognostic factors are represented by morphology (i.e., differentiation) and proliferation rate in terms of number of mitosis and proliferation index. Incomplete resection is the only widely accepted feature with negative prognostic significance. Prognostic models have been previously investigated to predict patients outcomes and tailor treatment. Filosso et al. (16) identified as negative prognostic factors male gender, age, previous malignancy, ECOG performance status, peripheral tumor, TNM stage. The presence of metastatic disease demonstrated to be the strongest negative prognostic factor (16, 17). In a large, single-center series of metastatic BC, age, bone metastases, liver metastases and Ki-67 index significantly correlated with prognosis (33). Few studies investigated prognostic indexes capable of estimating survival in bronchial NEN. Pusceddu et al. identified the NEP-Score, that was found to be useful to stratify survival probability both in very heterogeneous patients’ groups (24) and in a homogeneous cohort of stage IV entero-pancreatic NEN (25). Our present study demonstrated the validity of the NEP-Score in an independent series of 64 BC patients (7 ABC and 57 TBC) with very long follow-up. We indeed found that the NEP-T score, that takes into account the appearance of metastases during follow-up, was significantly associated with survival. In our series we evaluated the timing of metachronous metastases, 62.5% of which appeared after 24 months and 43.7% after 72 months, in keeping with previous findings (33) and supporting the indication for a long follow-up. We identified a NEP-T score threshold that could be useful to estimate OS. However, the limitation of this approach is represented by the need of a long follow-up of at least 24 months to calculate NEP-T score. On the other hand, a score that could be calculated at diagnosis, such as the NEP-D, could be more useful. Indeed, we found that a specific NEP-D threshold is associated with a higher OS, despite its reduced performance in predicting patients’ outcome in our settings. In particular, the identified NEP-D threshold was not able to predict 5 years survival and PFS, probably due to the small sample size. On the other hand, we found that NEP-D was significantly higher in male (but not in female) patients who were dead at EOF, suggesting that this score could be useful to predict prognosis in males. In addition, males showed higher NEP-T scores, more frequent relapses, a greater increase in Delta NEP and higher mortality for BC as compared to females, further supporting male gender as an independent negative prognostic factor, in keeping with previous reports (18, 19).

Delta NEP score may offer more indications, since it measures the development of metastases, with a different score depending on the timing of metastases appearance (before or after 24 months from diagnosis). The evidence that this score is significantly higher in patients dead as compared to those alive at EOF suggests that development of metastases, specially extrathoracic, profoundly influences survival in BC patients. Finally, we found that relapse rate was 2.5 fold higher in ABC as compared to TBC, confirming tumor differentiation as an important prognostic factor, in agreement with previous reports (18, 19).

Our study has some limitations, mainly represented by the small sample size and the low number of patients with ABC. Moreover, the influence of medical treatment outcome and therapeutic advances developed during the last years (29, 32, 33) were not taken into account. However, this first study opens the way to a validation study of the promising NEP-D score on several and larger cohorts of patients. Indeed, validation of this score in blarger BC cohorts, in a multicenter setting, is necessary in order to provide a better estimate of the NEP-D threshold, possibly contributing to improve patient’s management and quality of life.

## Data Availability Statement

The raw data supporting the conclusions of this article will be made available by the authors, without undue reservation.

## Ethics Statement

The studies involving human participants were reviewed and approved by Local Ethics Committee (Comitato Etico Indipendente di Area Vasta Emilia Centro, CE-AVEC, at the Policlinico S. Orsola-Malpighi in Bologna). Written informed consent for participation was not required for this study in accordance with the national legislation and the institutional requirements.

## Author Contributions

Conceptualization, MZ and MT. Methodology, PB. Investigation, MA, PF, RG, and AC. Writing—original draft preparation, MZ and MT. Writing—review and editing, IG and EG. Project administration, IG. Supervision and funding acquisition, MZ. All authors contributed to the article and approved the submitted version.

## Funding

This research was partially funded by the Italian Ministry of Education, University and Research fund (PRIN 2017Z3N3YC).

## Conflict of Interest

The authors declare that the research was conducted in the absence of any commercial or financial relationships that could be construed as a potential conflict of interest.
